# Perceptions of Private Medical Practitioners on Tuberculosis Notification: A Study from Chennai, South India

**DOI:** 10.1371/journal.pone.0147579

**Published:** 2016-01-28

**Authors:** Beena Elizabeth Thomas, Banurekha Velayutham, Kannan Thiruvengadam, Dina Nair, Sukendu Bikas Barman, Lavanya Jayabal, Senthanro Ovung, Soumya Swaminathan

**Affiliations:** 1 National Institute for Research in Tuberculosis (formerly Tuberculosis Research Centre), ICMR, Chennai, India; 2 National Institute for Research in Tribal Health, ICMR, Jabalpur, India; 3 Corporation of Chennai, Chennai, India; 4 Department of Health Research & Indian Council of Medical Research, New Delhi, India; McGill University, CANADA

## Abstract

**Background:**

The Government of India declared TB as a notifiable disease in 2012. There is a paucity of information on the government's mandatory TB notification order from the perspective of private medical practitioners (PPs).

**Objective:**

To understand the awareness, perception and barriers on TB notification among PPs in Chennai, India.

**Methods:**

Total of 190 PPs were approached in their clinics by trained field staff who collected data using a semi-structured and pre-coded questionnaire after getting informed consent. The data collected included PPs' specialization, TB management practices, awareness about the TB notification order, barriers in its implementation and their suggestions to improve notification.

**Results:**

Of 190 PPs from varied specializations, 138 (73%) had diagnosed TB cases in the prior three months, of whom 78% referred these patients to government facilities. Of 138 PPs, 73% were aware of the order on mandatory TB notification, of whom 46 (33%) had ever notified a TB case. Of 120 PPs, 63% reported reasons for not notifying TB cases. The main reasons reported for not notifying were lack of time (50%), concerns regarding patients' confidentiality (24%) and fear of offending patients (11%). Of 145 PPs, 76% provided feedback about information they felt uncomfortable reporting during notification. PPs felt most uncomfortable reporting patient's government-issued Aadhar number (77%), followed by patient's phone number (37%) and residential address (26%). The preferred means of notification was through mobile phone communication (24%), SMS (18%) and e-mail (17%).

**Conclusion:**

This study highlights that one-fourth of PPs were not aware of the TB notification order and not all those who were aware were notifying. While it is important to sensitize PPs on the importance of TB notification it is also important to understand the barriers faced by PPs and to make the process user-friendly in order to increase TB notification.

## Introduction

India accounts for a quarter of the 8.6 million cases of tuberculosis (TB) that occur worldwide **[1]**. The Government of India’s Revised National TB Control Programme (RNTCP) has ensured treatment through Directly Observed Therapy (DOT) which has been decentralized and made available free of cost. Despite this advancement it has been reported that India accounts for more than one-quarter of the estimated TB cases (i.e., about 1 million cases) that do not get diagnosed or notified **[2].** A large proportion of patients with TB prefer to seek care in the private healthcare sector, which often serves as the first point of care before referral to treatment in the public sector **[3, 4, 5]**. It has been reported that 50% of the retreatment cases notified under RNTCP are treated in other sectors before reaching RNTCP **[6]**.

This calls for a large-scale engagement of the country’s massive private health facilities and private medical practitioners (PPs) especially with regard to notification of TB. Notification could accelerate timely initiation of TB treatment, and proper treatment management under the government TB control programme that may lead to better outcomes. In addition, improved notification from PPs would also assist policy makers in refining estimates of India’s TB burden, and enable informed decisions in scaling-up of TB control activities in the country **[7]**.

In this background, with the aim of improving the collection of patient care information, the Government of India declared TB as a notifiable disease in the year 2012; where in, all TB cases diagnosed are to be reported mandatorily to the public health authorities in a specified format **[8].** As per this order, all healthcare providers are required to notify every TB case to the local public health authorities every month in a standard format. To facilitate notification, RNTCP also had formulated a web-based application called NIKSHAY in May 2012 that is meant to be used by all health functionaries across the country to notify cases **[1]**.

In spite of these efforts, persuading these private providers to notify the TB cases seen by them continues to be challenging **[7, 9, 10]**. As per the TB India 2014 RNTCP Annual Status Report, the overall percentage of notification by PPs was 3.1% **[1]**. Inorder to improve TB notification, it remains imperative to understand the awareness and perceptions of PPs about TB notification. This will help in gaining insight into the barriers to notification and in providing recommendations on how to facilitate notification. The findings of this study will help to develop concrete intervention strategies to improve notification by private care providers.

## Methodology

This study was part of a larger pilot study (Mobile Interface in TB Notification-MITUN) to determine the usefulness and feasibility of a mobile phone voice based system for notification of TB cases by PPs. This study was conducted during September 2013 to October 2014 in Chennai, South India. The study was approved by the Institutional Ethics Committee of National Institute for Research in Tuberculosis (NIRT) vide IEC No. 2013009. Written informed consent was obtained from the study participants prior to participation in the study.

### Study procedure

The study procedure involved mapping of all PPs registered in the areas being covered by three Tuberculosis Units (TUs) namely Tondiarpet, Pulianthope and Thiru Vi Ka Nagar selected based on convenience. Each TU has the responsibility of covering a population of 50000. The PPs or private clinics included any health establishment where TB cases are treated or diagnosed clinically or radiologically, and where medical services are provided by single medical practitioners who hold at least a MBBS degree who had diagnosed and or initiated treatment for at least one TB patient in the last 6 months. These practitioners may or may not be attached to any hospital. We did not include those practitioners from other systems of medicine.

A self-administered questionnaire was filled-in by the PPs that included particulars related to their specialization, the number of TB cases they have diagnosed or treated in the past 6 months, their awareness on the government’s order for mandatory TB notification. PPs perceptions and experiences regarding TB notification were collected through multiple option questions, reasons preventing them from notifying TB patients and their suggestions for improving TB notification **(S1 Appendix)**.

### Data analysis

The responses to the questionnaires were double verified, entered and analysed using IBM SPSS Version 20.0. The descriptive statistics of the PPs’ responses to the questions were calculated.

## Results

A total of 266 PPs were approached to assess their willingness to be a part of the study. Among them, 190 were willing, provided their informed consent, and were enrolled into the study. The reasons given for non-participation in the study for the remaining 76 PPs were: not interested or unwilling (45%), busy (14%) and do not diagnose TB patients (14%). Five PPs refused to participate in the study because their clinic associations did not permit disclosure of patient-related details, despite the fact that our questionnaire did not collect any confidential patient information. The PPs included were from varied specializations, with the majority of them being from MBBS without specialization(36%), followed by pediatrics (15%), chest medicine (6%), orthopedics (6%) and gynecology (5%).

### Diagnosis of TB and referral of patients by PPs to Government facilities

Of the total 190 PPs, 138 (73%) had diagnosed a TB case in the past 6 months. Of the 138 PPs who had diagnosed TB cases, 107 (78%) referred patients to government facilities. The reasons for referral were mostly because the patients could not afford private care in 101 (74%), the availability of free drugs in government facilities in 66 (48%), accessibility in 41 (30%), and on patient’s request in 30 (22%).

### Awareness of mandatory TB notification order and notification

With regard to TB case notification, 138 PPs (73%) of the 190 were aware of the government’s order on mandatory TB notification, while 52 (27%) of them were not aware of it. Awareness of the government’s order for TB notification was highest among PPs specialized in chest medicine 11 (92%), followed by pediatrics 22 (79%), general medicine 53 (78%), and gynecology 5 (56%) **(Table 1)**. Out of the 190 PPs, 56 (30%) had notified TB cases, while 134 (70%) had never notified TB patients. A higher proportion of gynecologists (44%) have notified TB cases followed by general physicians (35%), orthopedists (27%), pediatricians (26%), and chest specialists (17%). Among the 138 PPs who were aware about the notification order, 46 (34%) had notified TB cases and 91 (66%) had not notified. Ninety three percent of the enrolled PPs said they were comfortable in notifying TB patients to the government.

**Table 1:** Practitioners’ specialization Vs. Awareness regarding Government’s order on mandatory TB notification.

Type of Specialist	Yes		No	
	No.	%	No.	%
General medicine (MBBS)	15	22	53	78
Chest medicine	1	8	11	92
Gynecology	4	44	5	56
Pediatrics	6	21	22	79
Orthopedics	6	55	5	46
Other	20	32	42	67
**Total**	**52**	**27**	**138**	**73**

### Reasons for not notifying TB

**Fig 1:**
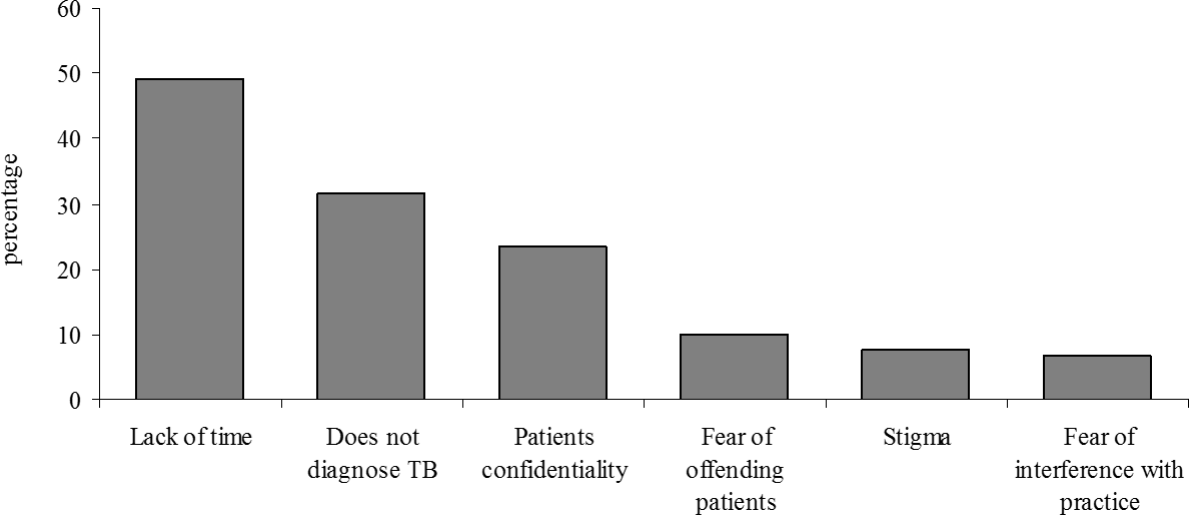
Possible reasons for preventing TB notification.

Of all PPs, 120 (63%) answered a question about reasons for not notifying TB cases. Of these 120 PPs, reasons reported for not notifying TB cases to government health facilities are lack of time 59 (50%), not diagnosing or treating TB cases 38 (32%), concerns regarding to patients’ confidentiality 28 (24%), fear of offending patients 12 (11%), stigma 9 (8%) and fear of interference with the practice 8 (7%) **(Fig 1).**

### Perceptions about information required in TB notification

All the 190 PPs who were enrolled to the study were asked about the information that was required for TB notification. We were able to elicit multiple responses from 145 PPs for these questions, which are presented in Table 2. The others were not able to spare their time, were unwilling to answer the questions, or could not be contacted despite many attempts.

**Table 2:** Information in the TB notification form that PPs were not comfortable reporting to the government (N = 145).

Personal identities	No.	%
Name	24	17
Father’s/Husband’s/Wife’s Name	29	20
Residential address	38	26
Aadhar No.	112	77
Phone No.	53	37
Age	6	4
Gender	10	7
**Treatment details**		
Investigations done	4	3
Date of diagnosis	4	3
Type of TB	6	4
Category of treatment	7	5
Date anti-TB therapy (ATT) was prescribed	1	1
Anti-TB drugs	1	1

We asked PPs whether there is information that they feel uncomfortable reporting to the government during the notification process; 145(76%) responded to this question With regard to patient-related details, 77% of the practitioners said that the most difficult information to provide was the government-issued Aadhar number, followed by the patient’s phone number (37%) and residential address (26%). Twenty percent were not comfortable in providing the name of the patient’s father or spouse (20%), patient’s name (17%), gender (7%) and age (4%). Other responses were related to the patients’ illness and treatment such as TB category (i.e., new or retreatment status), type of TB (pulmonary or extra-pulmonary), date of diagnosis, investigations, and the date that anti- tuberculosis therapy (ATT) was prescribed **(Table 2)**.

### Preferred means for improving TB

**Fig 2:**
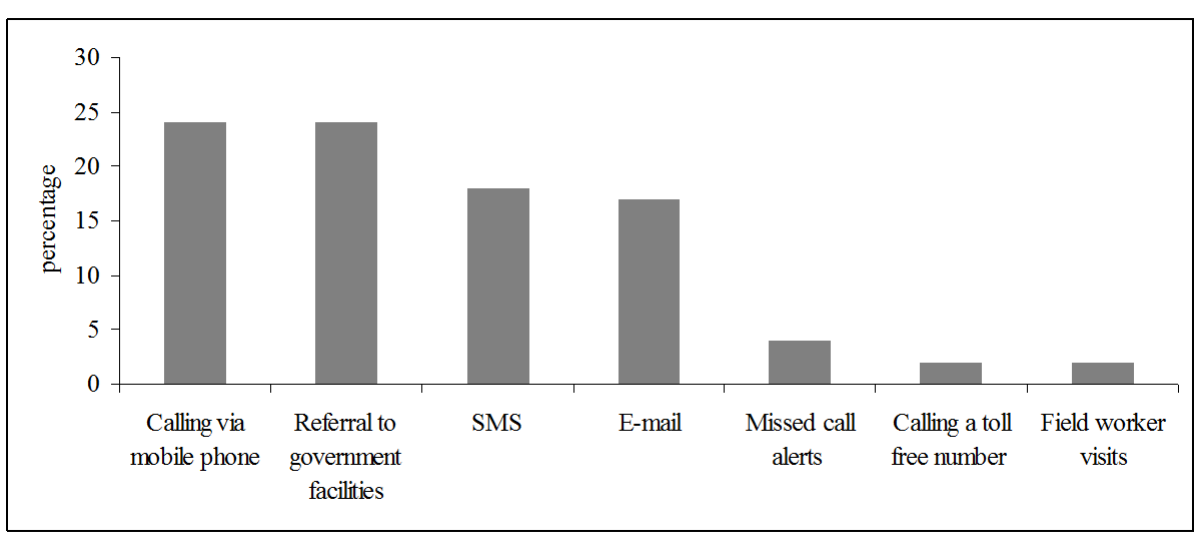
Preferred means of notification for improving TB notification.

Of the 190 PPs, we were able to elicit responses from 138 PPs on their preferred means of notification and suggestions to improve notification. The preferred means of notification were through mobile phone communication 33 (24%), SMS 25 (18%), e-mail 23 (17%), missed call alerts 6 (4%), calling a toll free number 3 (2%) and through field workers 3 (2%). Thirty-three (24%) of PPs said that they prefer referring patients to government facilities as a means of notification. Sixteen PPs (12%) expressed the need to create greater awareness on the mandatory TB notification **(Fig 2).**

## Discussion

In spite of the government order regarding mandatory TB notification being issued in 2012, only 72% of PPs were aware of this order. Awareness was highest among chest specialists (92%). This was corroborated with the findings of a study done in Alappuzha district of Kerala state that showed overall 88% of PPs and 100% of chest specialist were aware of the TB notification order **[11]**. However, it was a matter of concern that TB notification was lowest among chest specialists, with only 17% having ever notified a TB case. This finding suggests that awareness may not translate into increases in TB notification, especially among those who are likely to diagnose and notify a larger number of TB patients. Other studies on TB notification have reported difficulties in convincing PPs about the benefits of notifying TB patients to the public system, despite sensitization efforts **[12,13]**.

Previous research findings in India have indicated that, more than general awareness, there is need for educational inputs through regular training of PPs with regard to case notification **[14, 15].** Other interventional studies on case detection showed that it is possible to involve PPs in case notification by strengthening supervision of PPs and providing them with financial incentives **[16,17,18]**. This point to the need for further studies that assess the feasibility and impact of interventions that engage PPs to notify TB suspects to the public system. Time constraints, concerns about patient confidentiality and subsequent concern for patient-related stigma was reported as barriers that might prevent PPs from notifying TB patients to the government. Similarly, two studies carried out in India—one in Gujarat **[19]** and the other in Kerala **[20]—**reported that concerns regarding the violation of patient confidentiality and social stigma or discrimination against the patients were considered to be an impediment in notifying TB cases to the government. Besides this, PPs were concerned that notification might also interfere with their routine practice as it would attract scrutiny of their routine activities. Perhaps this reflects their feeling of some loss of independence in patient TB management.

All of the above calls for more effective, inclusive sensitization which needs to be PP friendly taking into consideration their concerns regarding notification and addressing these issues. This needs to be done through professional bodies like IMA (Indian Medical Association), NIMA (National Integrated Medical Association) and through various public-private intervention strategies to strengthen notification. This is further justified with few studies from India that reported that PPs with limited experience practicing in the public sector have inadequate training or knowledge about the RNTCP. The studies report that they are then less likely to get involved in TB case detection and notification **[12, 13, 14, 15, 17, 19].** These sensitization efforts need to also remind the PPs on aspects with regard to their concerns on breach of patient confidentiality. According to the Medical Council of India (MCI) code of Ethics–Rules and regulations 2002, Chapter 7, Point 7.14—“it is the duty of the registered medical practitioners to divulge this information to the authorized notification officials as regards communicable and notifiable diseases” **[1].**

It is also important to incorporate suggestions from PPs to strengthen notification. PPs indicated that, if given an option, their preferred means of notification would be through mobile phone communication or referral to government facilities. It is important to note that, currently, the only option available for PPs to notify cases is NIKSHAY, the government’s web-based application. The need for strengthening private public partnerships through effective communication strategies between both sectors becomes crucial to ensure that the patients PPs refer to public health facilities are notified. For this the public sector needs to have a mechanism of networking with PPs to ensure that patients referred to them are notified and also not duplicated in the system.

Suggestions for improvement also included allowing use of mobiles, simple text messages, toll free number with interactive voice response services, and missed call alerts to improve TB notification. These suggestions re-emphasize the recommendations of Nagaraja et.al, in 2014 that the TB control programme should provide simple and user- friendly information-communication-technology (ICT) platforms for notification **[12]**.

This study has few limitations. A self-administered questionnaire was used to collect information from PPs, which might have led to respondent bias, including over or under reporting of true TB notification. Furthermore, the questionnaire has limitation of not being able to gather adequate information as it depends on the time that the participant spends on completing it as compared to a face to face interview. This study was carried out in a metro city in South India and the results obtained may be unique to an urban setting. The sample size used for this study was small to generalize the findings in other settings. We had the limitation of not being able to include qualitative data using in-depth interviews which would have added more insight into the validity of the perceptions of private practitioners. However, due to time constraints and difficulties in getting private practitioners to participate in an interview was a challenge and could not be done. This needs to be further explored.

## Conclusion

In this study of PP notification practices in Chennai, we found that nearly one-fourth of all PPs were not aware of the government’s mandatory notification order. Of even greater concern is the fact that a large proportion of PPs who were aware of the order still fail to notify cases; this problem is especially acute among chest physicians, who probably see the highest number of TB patients. Inorder to facilitate effective TB notification by PPs it is important to evolve effective, user-friendly intervention strategies to strengthen notification. Our findings suggest that future interventions should provide PPs with multiple options for notifying cases especially via mobile phone—through direct calls, SMS, and mobile applications. In addition, it is crucial to address PPs concerns regarding maintaining patient confidentiality during the notification process. The study also endorses the need for effective communication strategies between the private and public sectors becomes so that TB patients referred to the government sector are not lost to the system or duplicated in the notification reporting system.

## Supporting Information

S1 Appendix Pre-Intervention Assessment Questionnaire. (DOCX)
