# Supplementary material for: Perceptions of Private Medical Practitioners on Tuberculosis Notification: A Study from Chennai, South India
Source: PLoS One. 2016 Jan 28;11(1):e0147579. doi: 10.1371/journal.pone.0147579 (PMC4731139; doi:10.1371/journal.pone.0147579)
Supplement: S1 Appendix — (DOCX) [file pone.0147579.s001.docx]

S1 Appendix

**The usefulness and feasibility of mobile phone based notification of tuberculosis by private health care providers – A pilot project (M**obile **I**nterphace in  **TU**berculosis **N**otification **- MITUN)**

**PRE-INTERVENTION ASSESSMENT QUESTIONNAIRE**

*(Version 1.1 dated 24 May, 2013)*

***Dear Doctor, We request you to kindly provide the following details***

**1. Name (with initials):**

**2. Degree / Diploma:**

**3. Medical Registration number:**

**4. Name of Medical Council:**

**5. Mobile No:**

**6. Type of Practice – General Practitioner Specialist**

**7. Place of Practice - Clinic Hospital Both**

**8. Location of practice:**

9. Approximate Number of TB cases diagnosed by you in the past 6 months: 10. Approximate Number of TB cases started on treatment by you in the past 6 months:

11. TB treatment that you prescribe- RNTCP (thrice-weekly) / Non-RNTCP (daily) 12. Are there patients that you refer to the Government health facilities?

Yes No 13. If yes, could you give us the reasons as to why you refer Cannot afford private care

On patients request Accessibility of Govt TB Rx Free drugs

Others

14. Are you aware of the Government order on making TB notification mandatory before our contact with you?

o Yes

o No

15. As a private medical practitioner, are you comfortable in notifying your TB patients to the Government?

o Yes

o No

16. Have you ever notified TB case before?

o Yes

o No

17. If yes, number of cases

18. If yes how:

19. What details have you provided about the patient?

20. Which do you feel is the convenient modality of notification of TB cases to the Government?

o By post

o By email / website o Via mobile phone o Others, specify:

**Signature of Dr. & Date**

***Thank you for the responses***
